# Subchondral Bone Relative Area and Density in Human Osteoarthritic Femoral Heads Assessed with Micro-CT before and after Mechanical Embedding of the Innovative Multi-Spiked Connecting Scaffold for Resurfacing THA Endoprostheses: A Pilot Study

**DOI:** 10.3390/jcm10132937

**Published:** 2021-06-30

**Authors:** Mikołaj Dąbrowski, Piotr Rogala, Ryszard Uklejewski, Adam Patalas, Mariusz Winiecki, Bartosz Gapiński

**Affiliations:** 1 Adult Spine Orthopaedics Department, W. Dega University Hospital, Poznan University of Medical Sciences, 28 Czerwca 1956 Street 135/147, 61-545 Poznan, Poland; 2 Institute of Health Sciences, Hipolit Cegielski State College of Higher Education, Card. Stefan Wyszyński Street 38, 62-200 Gniezno, Poland; gabinet.rogala@gmail.com; 3 Construction Materials and Biomaterials, Institute of Materials Engineering, Kazimierz Wielki University, Karola Chodkiewicza Street 30, 85-064 Bydgoszcz, Poland; uklejew@ukw.edu.pl (R.U.); winiecki@ukw.edu.pl (M.W.); 4 Division of Technology Design/Laboratory of Bone Implants Research and Design, Institute of Mechanical Technology, Poznan University of Technology, Piotrowo Street 3, 60-965 Poznan, Poland; adam.patalas@put.poznan.pl; 5 Division of Metrology and Measurement Systems, Institute of Mechanical Technology, Poznan University of Technology, Piotrowo 3, 60-965 Poznan, Poland; bartosz.gapinski@put.poznan.pl

**Keywords:** osteoarthritic hip, resected human femoral heads, subchondral trabecular bone relative area, subchondral trabecular bone density, micro-CT assessment, multi-spiked connecting scaffold (MSC-Scaffold), biomimetic fixation for resurfacing endoprostheses, surface replacement arthroplasty

## Abstract

The multi-spiked connecting scaffold (MSC-Scaffold) prototype is the essential innovation in the fixation of components of resurfacing total hip arthroplasty (THRA) endoprostheses in the subchondral trabecular bone. We conducted the computed micro-tomography (micro-CT) assessment of the subchondral trabecular bone microarchitecture before and after the MSC-Scaffold embedding in femoral heads removed during long-stem endoprosthesis total hip arthroplasty (THA) of different bone densities from 4 patients with hip osteoarthritis (OA). The embedding of the MSC-Scaffold in subchondral trabecular bone causes the change in its relative area (BA/TA, bone area/total area ratio) ranged from 18.2% to 24.7% (translating to the calculated density *ρ_B_* relative change 11.1–14.4%, and the compressive strength *S* relative change 75.3–122.7%) regardless of its initial density (before the MSC-Scaffold embedding). The densification of the trabecular microarchitecture of subchondral trabecular bone due to the MSC-Scaffold initial embedding gradually decreases with the increasing distance from the apexes of the MSC-Scaffold’s spikes while the spatial extent of this subchondral trabecular bone densification ranged from 1.5 to 2.5 mm (which is about half the height of the MSC-Scaffold’s spikes). It may be suggested, despite the limited number of examined femoral heads, that: (1) the magnitude of the effect of the MSC-Scaffold embedding on subchondral trabecular bone densification may be a factor contributing to the maintenance of the MSC-Scaffold also for decreased initial bone density values, (2) the deeper this effect of the subchondral trabecular bone densification, the better strength of subchondral trabecular bone, and as consequence, the better post-operative embedding of the MSC-Scaffold in the bone should be expected.

## 1. Introduction

Osteoarthritis (OA) is a common disease that mainly involves cartilage destruction, synovial inflammation, osteophyte formation, and subchondral trabecular bone sclerosis with progressive functional disability and reduced quality of life [1]. For OA of the hip, the main treatment option is total hip arthroplasty (THA), which is widely used, and there are many methods of fixation of implants and bearing surfaces [1,2]. Resurfacing THA (THRA) offers several potential advantages over long-stem endoprosthesis THA: preservation of the bone stock and near to physiologic bone biomechanics, improved stability, and excellent functional outcome [3,4,5].

The multi-spiked connecting scaffold (MSC-Scaffold) prototype is the essential innovation in the fixation of components of THRA endoprostheses in the periarticular trabecular bone. It was designed and manufactured in the selective laser melting (SLM) technology of Ti-alloy powder, developed in the mechanical laboratory and numerical studies, and verified in animals (10 swines) in our bioengineering-clinical research team guided by Uklejewski [6,7,8,9,10,11,12,13], based on the concept of such fixation proposed by Rogala [14,15].

The MSC-Scaffold’s spikes mimic the interdigitations (trabeculae) of the articular subchondral trabecular bone, which is a natural biostructure interfacing the articular cartilage with the periarticular trabecular bone. The system of subchondral trabecular bone interdigitations (or trabeculae) interpenetrating the trabeculae of periarticular cancellous bone provides a gradual, structural transition between the two different morphological biostructures—articular cartilage and periarticular trabecular bone of epiphysis [16,17]. The fixation procedure of THRA endoprosthesis with the biomimetic MSC-Scaffold proceeds in two steps as follows: (1) the surgical initial embedding of the endoprosthesis components into the periarticular trabecular bone to approximately half the height of the MSC-Scaffold spikes by the operating surgeon, and (2) the biologic fixation due to bone tissue ingrowth into the remaining inter-spikes region of the MSC-Scaffold during the post-operative rehabilitation of OA patient.

The already obtained and published research results [13] show that the proposed innovative fixation with the use of the MSC-Scaffold prototype allows for the entirely cementless and biomimetic fixation of the components of resurfacing endoprostheses in the periarticular trabecular bone with a satisfactory (good or very good) clinical stability. This biomimetic fixation, as the alternative for the contemporary standard fixation of the THRA components in bone with the use of cement [18,19,20], can be regarded as a promising breakthrough in advanced bone-implant interfacing in joint resurfacing endoprostheses.

For the appropriate bioengineering design of the innovative MSC-Scaffold prototype for human THRA endoprostheses that could be used in the surgical treatment of patients with OA, it is necessary to perform the computed micro-tomography (micro-CT) assessment of the subchondral trabecular bone relative area and density in the femoral heads resected from OA patients surgeried with THA, before and after mechanical embedding the MSC-Scaffold prototype into these heads.

The quantitative-CT (QCT) studies of the subchondral trabecular bone microstructure in the medial proximal tibia and femoral heads OA were carried out in [21,22,23,24]. The most common studies were to perform analysis of subchondral trabecular bone in patients with medial knee OA, to elucidate features of bone microstructure in OA, and to investigate relationships between bone microstructure and both stages of the disease and lower limb alignment [21]. The investigation of age- and gender-related changes of microarchitecture and bone remodeling in subchondral trabecular bone in OA has also been considered [23].

The micro-CT bone density assessment performed on animal (swine) fresh femoral heads published in [25] revealed that the embedding of the MSC-Scaffold prototype in the periarticular bone of femoral heads causes bone material densification under the embedded MSC-Scaffold that affects its mechanical properties. Therefore, it can be hypothesized: the densification of microarchitecture of subchondral trabecular bone of femoral heads from patients with OA due to the MSC-Scaffold embedding in femoral head specimens may also depend on the initial bone density in these femoral heads. The basic aim of the study was the micro-CT assessment of the subchondral trabecular bone relative area and density in the femoral heads removed during THA from OA patients carried out before and after mechanical embedding of the innovative MSC-Scaffold prototype for THRA endoprostheses into these heads. The specific aim of the study is to evaluate the influence of the initial surgical embedding of the MSC-Scaffold prototype for THRA endoprostheses on the change in the microstructure of the trabecular microarchitecture of the subchondral trabecular bone of femoral heads from OA patients, as possible, depending on the initial value of the subchondral trabecular bone relative area in these heads.

## 2. Materials and Methods

### 2.1. Patient Selection

All cases included in the study showed severe stages of primary OA in the hip joint and underwent THR. Exclusion criteria were secondary degenerative changes and hip fractures, history of cancer, liver or kidney failure.

This study was carried out in the frame of a research grant of the Poznan University of Medical Science, approved by the Bioethics Committee (Approval No. 146/2018).

### 2.2. Radiological and Pain Assessment 

All subjects included in this study were undergoing bilateral anteroposterior radiography of the hip. The orthopedist assessed the radiographs by using the Kellgren-Lawrence (KL) radiographic scale. Stage 2 is described as definite osteophyte formation with possible joint space narrowing, stage 3—multiple osteophytes, definite joint space narrowing, sclerosis, and possible bony deformity, and stage 4—large osteophytes, marked joint space narrowing, severe sclerosis, and definite bony deformity. Visual analog scale (VAS) score is used to measure the pain intensity of the hip joint. It has the range of 0–10, with 0 being no pain and 10 extreme pain.

### 2.3. Femoral Heads Sampling

All of the femoral heads were acquired intraoperatively from the patients undergoing THA for OA of the hip. The shape of the examined proximal ends of the femurs had been changed by the degenerative process. Bone tissue densification at the superolateral part of the femoral head and cysts in the femoral head and neck were present. Articular surfaces exhibited defects that were accentuated at the loading area. Articular cartilage was pathologically changed, softened, and disintegrated, with areas of local destruction due to subchondral congestion and blood vessel infiltration.

### 2.4. Femoral Head Specimens Preparation

The femoral head specimens after surgical resection were covered with a dressing soaked with saline solution (NaCl 0.9%) and packed in an airtight package to prevent them from drying. Subsequently, the bone specimens were transported to the test site in a portable refrigerator and were machined under constant water irrigation alignment of the femoral head-neck area and removal of a piece of cartilage around the top of the head (by face milling). Then, the bone specimens were immediately subjected to micro-CT imaging. The total time between femoral head specimens resection and the end of their micro-CT imaging was no longer than 6 h.

### 2.5. MSC-Scaffold Preprototypes

The MSC-Scaffold preprototypes were manufactured in the Selective Laser Melting (SLM) technology on the REALIZER II 250 SLM^®^ machine (MTT Technologies Group, Paderborn, Germany) of Ti6Al4V powder. Details regarding the design of the MSC-Scaffold CAD models, the applied SLM process parameters, and the SLM post-processing treatment are given in [25].

### 2.6. Micro-CT Examination

The micro-CT examination was performed on the micro-CT system (GE phoenix v|tome|x s240, Waygate Technologies, Wunstorf, Germany). The micro-CT system included the specialized device designed and manufactured by our research team [25]. This device enables the mechanical embedding of the MSC-Scaffold in the femoral head specimens directly inside the scanning chamber of the micro-CT scanner and the real-time X-ray scanning control of the embedding process. The scanning parameters used for the micro-CT scans were 25.3 W (230 kV/110 μA), exposition time for one picture was 250 milliseconds and the voxel size was 12.2 μm. The 0.25 mm brass filter was applied. The full scan time was approx. 20 min.

Following the micro-CT scanning of every femoral head specimen, the titanium MSC-Scaffold prototype was placed on the specimen’s top, and, applying the specialized device, the MSC-Scaffold embedding in the femoral head specimen was carried out with the speed of 0.1 mm/s to halfway up the spikes. The embedding process was controlled radiologically to achieve the proper depth of embedding. Afterward, the micro-CT scanning of the femoral head specimen was done again with the same scanning parameters.

### 2.7. Micro-CT 3D Reconstruction of Specimens

To accurately reconstruct the trabecular microarchitecture of subchondral trabecular bone in femoral head specimens, before and after the MSC-Scaffold prototype mechanical embedding to perform the qualitative and quantitative micro-CT analyses of this microarchitecture, the following image processing steps (necessary for the acquired projection data sets) were done: 3D image reconstruction of femoral head specimens, elimination of imaging artifacts, segmentation of images.

In the reconstructed bone-implant specimens, different elements such as the Ti-alloy MSC-Scaffold prototype, bone trabeculae, and soft tissues between trabeculae (including bone marrow) were identified based on their radiological density. Since the resolution of micro-CT is unable to distinguish the intermediate phase and the sub-resolution pores and voxels containing multiple phases are misclassified [26], additional correction with a thresholding method was applied. This method assigns the voxels of different grayscale to the specified phase based on the selected threshold, which increases control of determining the boundaries of distinguished elements. In the case of the micro-CT images of porous materials, such an approach is widely applied [27,28,29].

The same identification procedure was applied for the femoral head specimens before embedding the MSC-Scaffold, following the same criteria regarding the identification of only the two radiological phases: bone trabeculae, and soft tissues between trabeculae.

**Figure 1:**
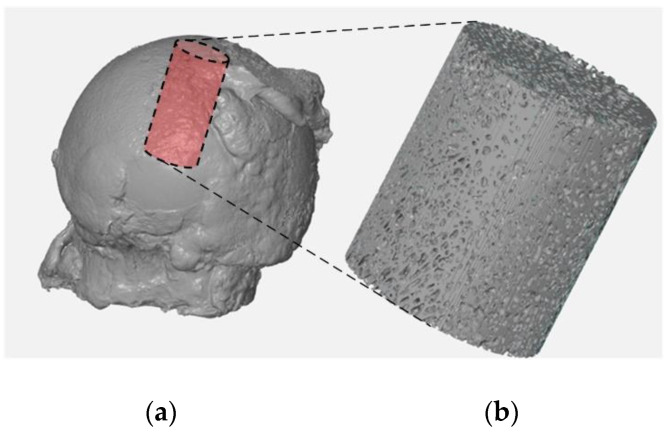
Micro-CT reconstruction of the femoral head specimen (**a**) and the digitally extracted cylinder-shaped bone sample (**b**) for further micro-CT evaluation of bone trabecular microarchitecture and density.

**Figure 2:**
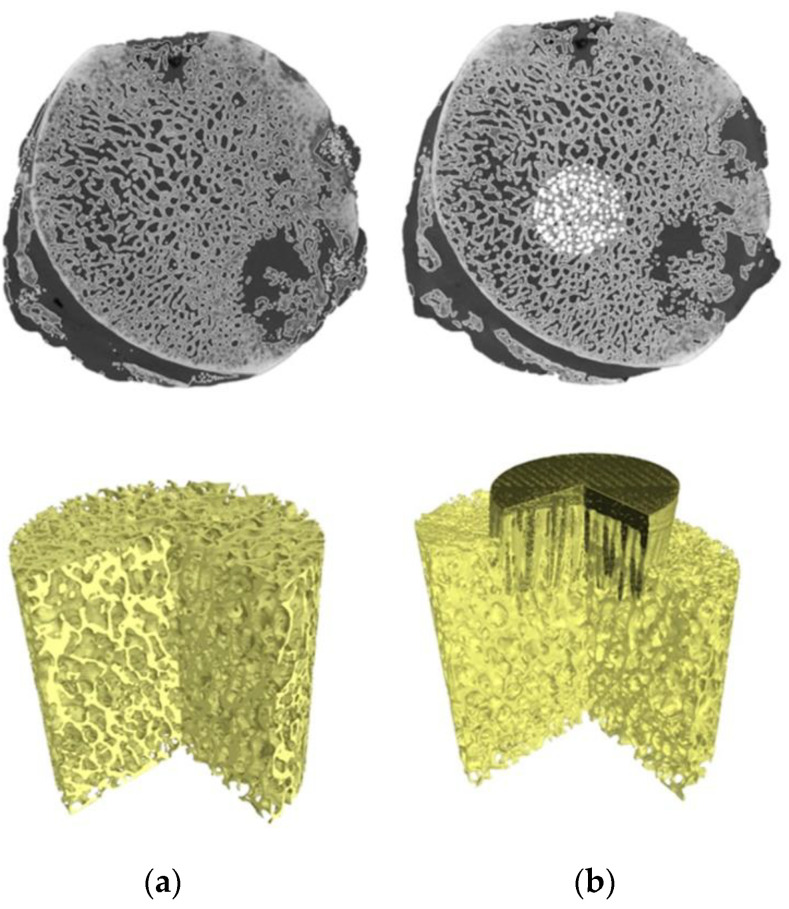
Micro-CT 3D reconstruction of femoral head bone samples: (**a**) before and (**b**) after embedding of MSC-Scaffold prototype.

For the reconstructed femoral head specimens before and after mechanical embedding of the MSC-Scaffold prototype, the same coordinate systems were established and the datasets were compared using the professional software tool (Volume Graphics 2.2 software, Heidelberg, Germany). The cylinder-shaped fragments of the femoral head reconstructions located in the region of the MSC-Scaffold prototype embedding site have been digitally extracted. The bone cylinder axis was consistent with the direction of the resultant compression force acting on the femoral head during functional loading of the leg [30,31]. An exemplary image of the femoral head 3D-reconstruction is shown in Figure 1. The exemplary micro-CT reconstructed cross-sections of femoral head samples before and after embedding of MSC-Scaffold are shown in Figure 2.

Then, for each digitally extracted cylinder-shaped bone sample reconstructions the reference cross-sections were assigned to analyze. The reference cross-sections were spaced 0.5 mm from each other and the reference plane was established as the plane tangent to the spikes apexes of the embedded MSC-Scaffold prototype. For the cylinder-shaped micro-CT reconstructions of femoral head bone before the MSC-Scaffold prototype embedding, the reference plane was set at the same level relative to the top of the cylinder. In each micro-CT reconstructed cross-section, the radiological compartments representing bone trabeculae and inter-trabecular regions (pore spaces) with soft tissues were identified.

### 2.8. Determining of Subchondral Trabecular Bone Relative Area, Relative Density, and Compression Strength

The subchondral trabecular bone relative area was determined as the ratio of subchondral trabecular bone area to total area (BA/TA). Measurements were performed on binarized images (cf. [32]). As it is known for a porous bone [33,34], the mean value of the bone relative area (mean BA/TA) is equal to the bone relative volume (BV/TV) value. 

The volumetric subchondral trabecular bone density *ρ_B_* was calculated using the following formula [35,36]:

*ρ_B_* = *φ* · *ρ_T_* + (1 − *φ*) · *ρ_W_* (1)

where:

*ρ_T_*—the volumetric density of bone trabeculae (approximately equal to the cortical bone volumetric density, i.e., 1.85 g/cm^3^) [35,37];

*ρ_W_*—soft tissues volumetric density (approximately equal to that of water, i.e., 1.00 g/cm^3^);

*φ*—subchondral trabecular bone relative volume (BV/TV = mean BA/TA);

(1 − *φ*)—inter-trabecular soft tissue relative volume.

Then, to determine the changes in mechanical strength of bone caused by the MSC-Scaffold embedding, the compressive strength *S* values of subchondral trabecular bone of examined femoral heads in the regions of interest before and after embedding were calculated using the known empirical formula [38,39]:

*S* = 25 · (*ρ_A_*) ^1.8^ (2)

where: 

*S*—bone compressive strength [MPa]

*ρ_A_*—bone apparent density (= *φ* · *ρ_T_*) [g/cm^3^].

## 3. Results

### 3.1. Characteristics of the Patients 

**Figure 3:**
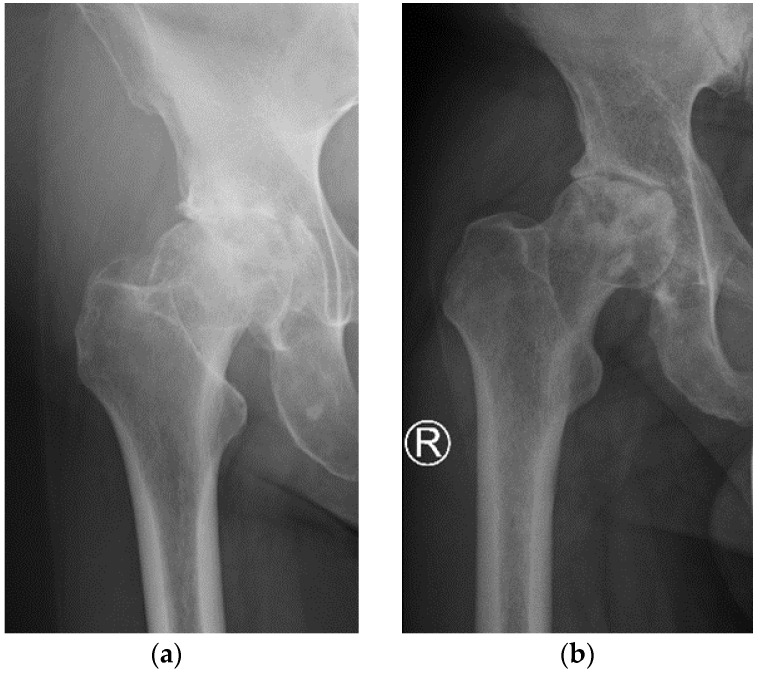
Pre-operative AP X-Ray right hip joints of two patients: (**a**) patient 2; (**b**) patient 4.

The detailed characteristics of the patients are given in Table 1. The study included 2 women and 2 men with severe stages of primary OA. All patients underwent THR surgery on the right hip. The pre-operative AP X-Ray right hip joints of two patients are presented in Figure 3. The average age of patients at the time of operation was 61.2 (range 51–72 years). The mean pain intensity of the patients on the VAS scale was 7.0. In the preoperative X-ray AP of the hip joint, two patients had grade 4 of the hip OA (KL4), and two patients had grade 3 (KL3). Patients had hip flexion contracture with limited flexion up to 80–90 and internal rotation to 5 degrees, accompanied by pain. The patient walked with a limp on the right lower limb. It was the first total hip replacement (THR) for all patients. Men had arterial hypertension and diabetes, and women had no comorbidities.

### 3.2. Subchondral Trabecular Bone Relative Surface before and after MSC-Scaffold Prototype Embedding 

**Figure 4:**
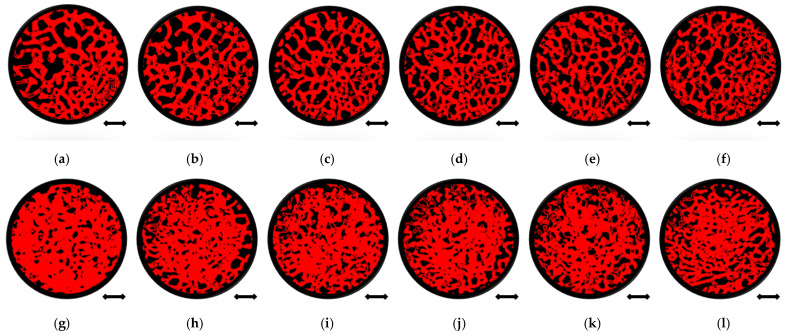
Quantitative micro-CT analysis of femoral head bone samples microarchitecture before and after mechanical embedding of the MSC-Scaffold prototype. Views of bone samples cross-sections below of the reference plane: before the MSC-Scaffold embedding (**a**) 0 mm, (**b**) 0.5 mm, (**c**) 1 mm, (**d**) 1.5 mm, (**e**) 2 mm, (**f**) 2.5 mm, and after MSC-Scaffold embedding (**g**) 0 mm, (**h**) 0.5 mm, (**i**) 1 mm, (**j**) 1.5 mm, (**k**) 2 mm, (**l**) 2.5 mm; bar = 2 mm.

The exemplary micro-CT cross-sections (slices) for patient 1 are shown in Figure 4, where femoral head bone fragments from a similar location before and after the MSC-Scaffold prototype embedding were juxtaposed for comparison.

The comparison of the bone sample’s cross-sections before (Figure 4a–f) and after (Figure 4g–l) mechanical embedding of the MSC-Scaffold prototype reveals changes in the microarchitecture of the subchondral trabecular bone due to the MSC-Scaffold embedding. Compared to each other the bone sample’s cross-sections after mechanical embedding of the MSC-Scaffold referring to subsequent levels below the reference plane (Figure 4g–l), the decrease in the extent of the densified subchondral trabecular bone area can be observed with increasing distance from the reference plane.

**Figure 5:**
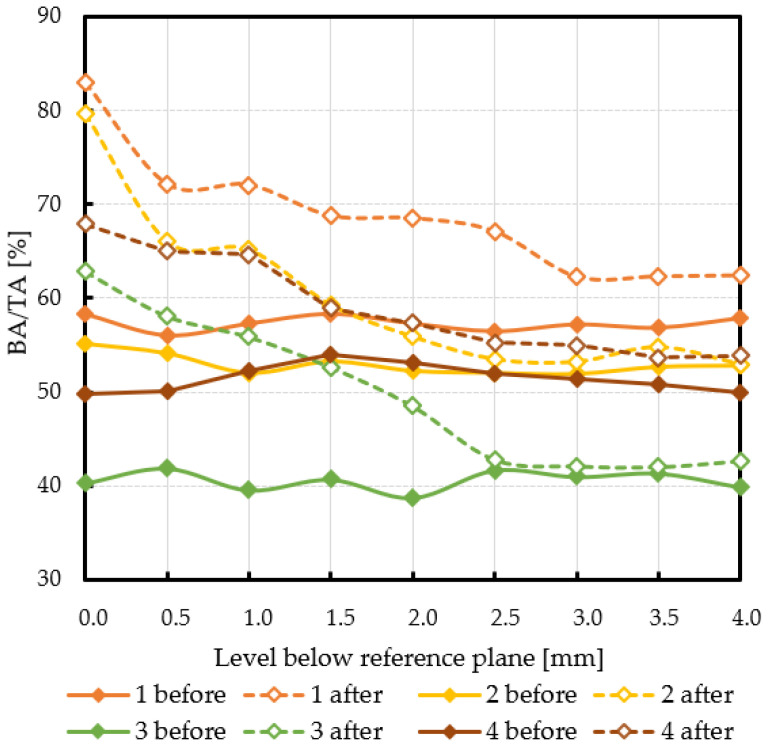
The subchondral trabecular bone relative area (BA/TA, bone area/total area ratio) values in micro-CT analyzed femoral heads’ samples removed during THA from OA patients before and after MSC-Scaffold prototype embedding, on various section levels below the reference plane.

**Figure 6:**
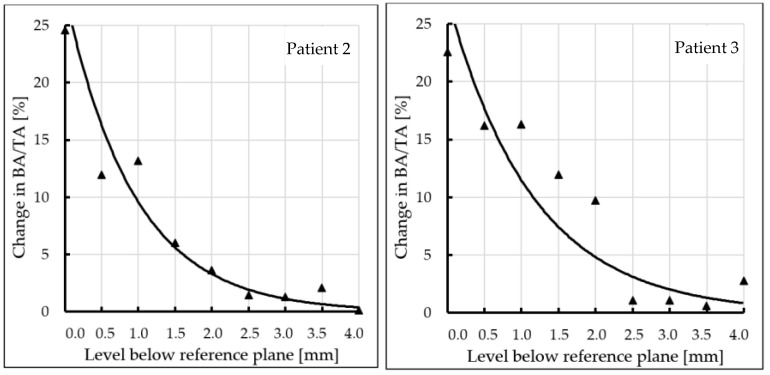
Changes in subchondral trabecular bone relative area (BA/TA, bone area/total area ratio) values in analyzed femoral head samples at different levels below the reference plane.

**Figure 7:**
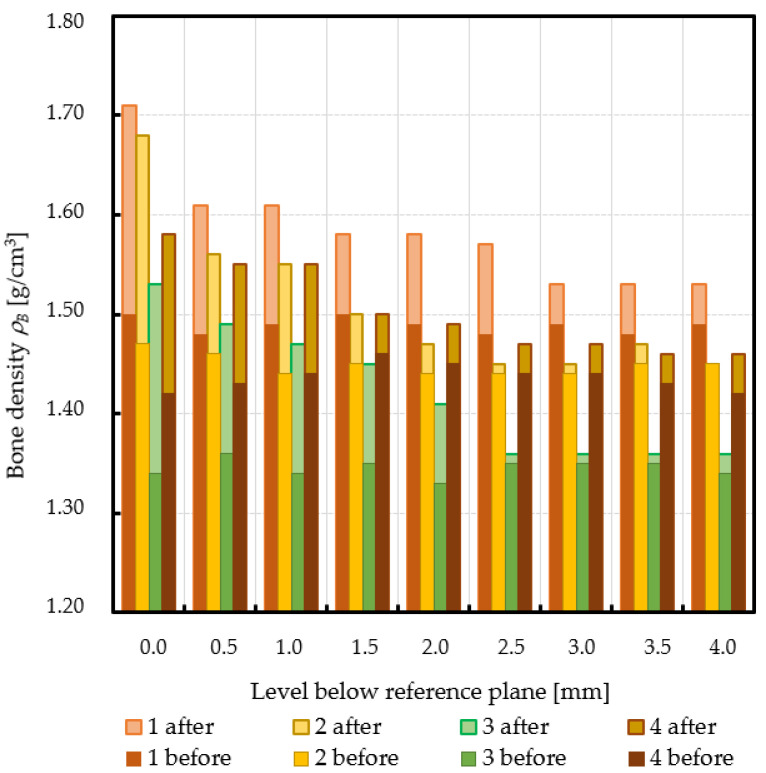
Changes in subchondral trabecular bone volumetric density values in analyzed femoral head samples at different levels below the reference plane.

**Figure 8:**
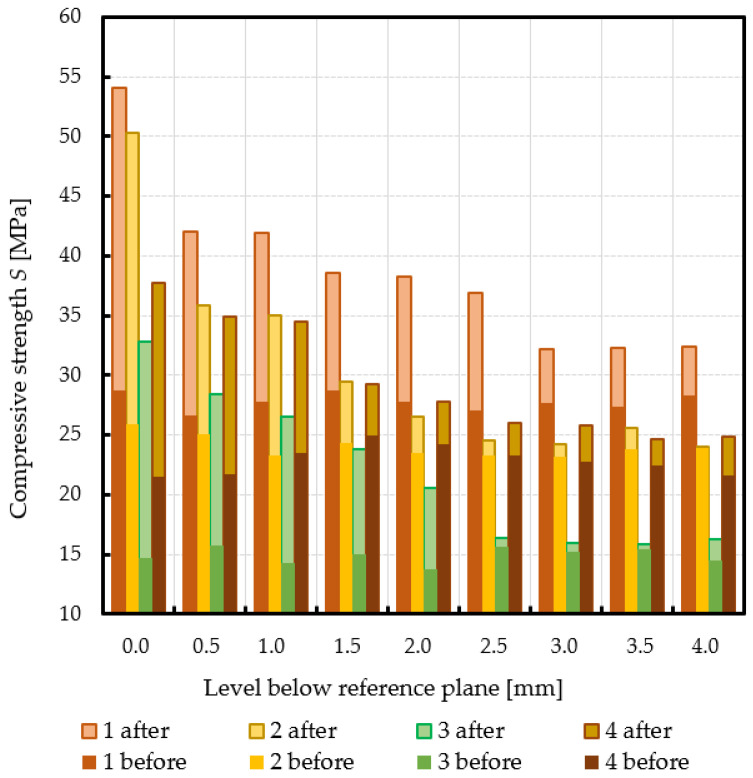
Changes in subchondral trabecular bone compressive strength values in analyzed femoral head samples due to the MSC-Scaffold initial embedding at different levels below the reference plane.

Subchondral trabecular bone relative area (BA/TA) values in femoral heads from patients with OA measured in micro-CT before and after embedding of the MSC-Scaffold, the percentage point change in BA/TA values, the calculated corresponding subchondral trabecular bone densities and subchondral trabecular bone compressive strength values (before and after the MSC-Scaffold embedding) are presented in Figure 5, Figure 6, Figure 7, and Figure 8, respectively. All the measured and calculated data are given in Table 2.

The subchondral trabecular bone BA/TA values in micro-CT analyzed femoral heads’ samples before the MSC-Scaffold embedding varied from 38.7% for patient 3 to 58.4% for patient 1, but did not change significantly within the volume of each analyzed sample (the changes in BA/TA values did not exceed 1.5% in each sample). The calculated mean subchondral trabecular bone density *ρ_B_* and mean subchondral trabecular bone compressive strength *S* before the MSC-Scaffold embedding varied from 1.34 ± 0.01 g/cm^3^ and 14.9 ± 0.7 MPa for patient 3, respectively to 1.49 ± 0.01 g/cm^3^ and 27.8 ± 0.7 MPa for patient 1.

After the MSC-Scaffold embedding, the BA/TA values were significantly higher and varied from 62.4% for patient 3 to 83.0% for patient 1 at the level just below the reference plane, while the corresponding calculated subchondral trabecular bone density *ρ_B_* for the reference plane ranged from 1.53 g/cm^3^ to 1.71 g/cm^3^, and the predicted subchondral trabecular bone compressive strength ranged from 32.8 MPa to 54.1 MPa. The subchondral trabecular bone density *ρ_B_* relative change at the level below the reference plane was from 11.1% to 14.4%, while the subchondral trabecular bone compressive strength relative change at the level below the reference plane was from 75.3% to 88.6%.

The BA/TA values then decreased at the next levels and the decrease rate varied from 4.2 p.p. to 18.3 p.p. per 1 mm, while the subchondral trabecular bone density *ρ_B_* relative change and the subchondral trabecular bone compressive strength relative change varied from 6.1% to 10.0% and from 34.3% to 86.2%, respectively. Such a decrease rate can be observed on average up to 2.0 mm depth below the reference plane. The BA/TA values decrease rates are different in each analyzed femoral head sample and the curves of best fit were estimated for each patient individually (Figure 6). As it can be seen in Figure 6, the particular best-fit curves of BA/TA decrease reach a plateau at the level from 1.5 to 3.0 mm below the reference plane. Below these levels the changes in subchondral trabecular bone BA/TA, subchondral trabecular bone density *ρ_B_* relative change, and subchondral trabecular bone compressive strength *S* are minor below 5 p.p., up to 3.1%, and up to 17.8%, respectively. Based on this the spatial extent of subchondral trabecular bone densification due to the MSC-Scaffold prototype initial embedding was established and it was marked with an asterisk in Table 2.

## 4. Discussion

Our innovative MSC-Scaffold prototype provides a new kind of biomimetic entire cementless fixation method for resurfacing THA endoprostheses components. Its embedding in bone is an original approach, thus there are no published other studies regarding this, or other similar surgical ways of surgical embedding, and there is furthermore no similar kind of test reflecting this embedding process to discuss. Therefore, it is not possible to compare the results obtained in the present study with the results obtained by other authors.

The mechanical embedding of the MSC-Scaffold prototype causes changes in the microarchitecture of the subchondral trabecular bone. After the MSC-Scaffold embedding, the BA/TA values measured in the subchondral trabecular bone at the level just below the spikes were significantly higher (from 18.2 to 24.7%). This change translates to the increase of the calculated subchondral bone density *ρ_B_* (relative change from 11.1 to 14.4%) and the predicted compressive strength *S* (relative change from 75.3 to 122.7%), regardless of the initial density of the bone (before the embedding). It was found that the subchondral trabecular bone densification due to the MSC-Scaffold initial embedding gradually decreases with the increasing distance from the apexes of the MSC-Scaffold’s spikes, while the spatial extent of the subchondral trabecular bone densification ranged from 1.5 to 2.5 mm. Beyond this densified bone material region the changes in subchondral trabecular bone BA/TA, subchondral trabecular bone density *ρ_B_* relative change, and subchondral trabecular bone compressive strength *S* are minor—below 5 p.p., up to 3.1%, and up to 17.8%, respectively.

It should be underlined that the BA/TA values of the subchondral trabecular bone of the femoral head specimens presented in Table 2 are mean values, calculated from the three BA/TA measurements: for the central slice at a given level below the reference plane and the two adjacent slices (placed at the distance of 0.5 mm from this central slice); these BA/TA values are, as it is known [34,40], equal to the bone relative volume BV/TV values (at a given level below the reference plane). The here assessed with micro-CT subchondral trabecular bone relative area (BA/TA) values in femoral head specimens removed during THA from OA patients are between 38.7% and 58.4% and correspond with the data provided by other authors research reported in the literature—e.g.: 60.2% [23,24], 32.7–59.7% [41]. The values of calculated mean subchondral trabecular bone density *ρ_B_* also correspond with data given by other authors [35,36].

It is known that the volumetric density of subchondral trabecular bone can be treated as the key parameter because its values can be assessed in human persons in vivo in various localizations in the skeleton e.g., utilizing the quantitative computed tomography (QCT, pQCT). 

Since the value of bone strength cannot be measured in patients in vivo, therefore, the empirical relationships linking the bone density with its biomechanical properties (such as bone strength, or Young’s elastic modulus) have been determined in laboratory biomechanical examinations carried out on bone preparations from different anatomical locations in the skeleton. A review of published scientific papers on this issue is included in the work by Fleps et al. [39].

Owing to these laboratory biomechanical studies and the published results of these studies, one can now use these known empirical relationships and calculate (with acceptable accuracy) the values of specific bone biomechanical parameters based on the values of bone density measured at various anatomical locations in the skeleton in a given patient. Thus, we have used the empirical formula (2) (see Section 2.8) established in [38,39] for the determination of the values of subchondral trabecular bone strength based on assessed values of subchondral trabecular bone density in examined femoral heads.

The changes in the subchondral trabecular bone relative area BA/TA values before and after embedding of the MSC-Scaffold prototype differed between analyzed femoral head specimens from OA patients. 

In patient 1 (male, 72 years old), the BA/TA value before the MSC-Scaffold embedding was about 56.1–58.4% (mean 57.3 ± 0.8%), the subchondral trabecular bone density *ρ_B_* was 1.49 ± 0.01 g/cm^3^, and the subchondral trabecular bone compressive strength *S* was 27.8 ± 0.7 MPa. After the MSC-Scaffold embedding, the subchondral trabecular bone relative area BA/TA at the level just below the reference plane was 83.0% (the corresponding calculated subchondral trabecular bone density was 1.71 g/cm^3^ and the subchondral trabecular bone compressive strength was 54.1 MPa), so the change between the highest BA/TA values (at the level just below the reference plane) was 24.7 p.p. The highest calculated relative densification of subchondral trabecular bone at this level was 14.3%, while the highest relative increase of the compressive strength was 88.6%. The changes in BA/TA value for patient 1 (evaluated from the best fit curve—Figure 6) then decreased to 15.0% at the level 1 mm (decrease rate 9.4 p.p. per 1 mm), reaching ca. 10.0% at the level 2 mm (decrease rate 5.0 p.p. per 1 mm), then ca. 3.5% at 3 mm (decrease rate 4 p.p. per 1 mm), and 4.5% at 4 mm (decrease rate 1.5 p.p. per 1 mm), respectively. The subchondral trabecular bone relative densification at the levels 0.5 mm and 1 mm was by 9.0% and 8.3%, respectively, and at the levels from 2.5 mm to 1.5 mm it was ca. 6.0%. The relative change in the compressive strength of subchondral trabecular bone at the levels 0.5 mm and 1 mm was by 57.5% and 51.0%, respectively, and at the levels from 2.5 mm to 1.5 mm it was ca. 36.0%. We can observe that in this patient the spatial extent of subchondral trabecular bone densification due to the MSC-Scaffold prototype initial embedding was 2.5–3.0 mm. Below this level, the relative densification of subchondral trabecular bone did not exceed 3%. The duration of symptoms was longer than in other patients and was 5 years X-ray indicated the radiological changes to the 3. stage in the KL scale. In severe pain (VAS 8) the patient moved with the support of a walker. OA of the hip was also found on the left side.

The BA/TA value in patient 2 (female, 54 years old) before the MSC-Scaffold embedding was approx. 51.9–55.1% (mean 52.9 ± 1.1%) and was slightly lower than in the previous patient. It translated for lower subchondral trabecular bone density *ρ_B_*—1.45 ± 0.01 g/cm^3^, and lower subchondral trabecular bone compressive strength *S*—24.0 ± 0.9 MPa. After the MSC-Scaffold embedding the BA/TA, at the level just below the reference plane was 79.7% (the corresponding calculated subchondral trabecular bone density was 1.68 g/cm^3^ and the subchondral trabecular bone compressive strength was 50.3 MPa), so the change between the highest BA/TA values (at the level just below the reference plane) was 24.6 p.p. The highest calculated relative densification of subchondral trabecular bone at this level was 14.4%, while the highest relative increase of the compressive strength was 94.3%. The changes in BA/TA value for patient 2 (evaluated from the best fit curve—Figure 6) then decreased to 10.0% at the level 1 mm (decrease rate 14.6 p.p. per 1 mm), reaching ca. 3.7% at the level 2 mm (decrease rate 6.3 p.p. per 1 mm), and ca. 1.3% at 3 mm (decrease rate 2.4 p.p. per 1 mm), respectively. The subchondral trabecular bone relative densification at the levels 0.5 mm and 1 mm was by 6.9% and 7.5%, respectively, and it was 3.3% at the level 1.5 mm. The relative change in the compressive strength of subchondral trabecular bone at the levels 0.5 mm and 1 mm was by 43.2% and 50.1%, respectively, and it was 21.3% at the level 1.5 mm. In this patient, the spatial extent of subchondral trabecular bone densification below the embedded MSC-Scaffold was 2.0–2.5 mm. The relative densification of subchondral trabecular bone out of this region was at the level of circa 0.5%, but it did not exceed 2%. The duration of symptoms was short, 12 months. X-ray indicated the 4 stage in the KL scale. Reported pain at VAS 7 and moving independently without elbow crutches. OA of both hip joints concerned.

The BA/TA value in patient 3 (female, 68) before embedding of the MSC-Scaffold was the lowest among the studied group, and it was in the range of 38.7–41.9% (mean 40.6 ± 1.0%). The subchondral trabecular bone density *ρ_B_* was 1.34 ± 0.01 g/cm^3^, and the subchondral trabecular bone compressive strength *S* was 14.9 ± 0.7 MPa. After the MSC-Scaffold embedding BA/TA at the level just below the reference plane was 62.9% (the corresponding calculated subchondral trabecular bone density was 1.53 g/cm^3^ and the subchondral trabecular bone compressive strength was 32.8 MPa), so the change between the highest BA/TA values (at the level just below the reference plane) was 22.6 p.p. The highest calculated relative densification of subchondral trabecular bone at this level was 14.0%, while the highest relative increase of the compressive strength was 122.7%. The changes in BA/TA value for patient 3 (evaluated from the best fit curve Figure 6) then decreased to ca. 12.0% at the level 1 mm (decrease rate 10.57 p.p. per 1 mm), reaching 5.0% at the level 2 mm (decrease rate 7.0 p.p. per 1 mm), and ca. 2.5% at 3 mm (decrease rate 2.5 p.p. per 1 mm), respectively. The subchondral trabecular bone relative densification at the levels 0.5 mm and 1 mm was by ca. 10%, at the levels from 2.0 mm to 1.5 mm it was ca. 7%. The relative change in the compressive strength of subchondral trabecular bone at the levels 0.5 mm and 1 mm was by ca. 80%, at the levels from 2.0 mm to 1.5 mm it was ca. 50%. The spatial extent of subchondral trabecular bone densification due to the MSC-Scaffold prototype initial embedding was here 2.0–2.5 mm. The relative densification of subchondral trabecular bone out of this region was at the level of circa 0.5%, but it did not exceed 1.6%, while the subchondral trabecular bone compressive strength was circa 4.8%. In this patient, the duration of symptoms was intermediate, 2 years. X-ray indicated the 4 stage of the KL scale. Pain intensity was VAS 7, and the patient moved with elbow crutches. OA of both hip joints concerned. The baseline bone density in this patient was the lowest in the whole group. Li et al. reported BV/TV parameters for patients with osteoporosis 20.50% (16.72–24.28%) [24]. BA/TA value of about 40% indicates intermediate values and may indicate osteopenia. Additionally, age and gender risk factors may indicate a decrease in bone density. Initially, the lower bone density before the MSC-Scaffold embedding resulted in a density below it (14%), similar to that of patients with higher bone density. Moreover, the depth of impact of the MSC-Scaffold was approx. 2.0–2.5 mm and was similar to that of patients with good baseline bone density.

In the last patient, patient 4 (male, 51 years old), the subchondral trabecular bone relative area BA/TA value before the MSC-Scaffold embedding was intermediate in the study group, i.e., it was in the range of 49.7–54.0% (mean 51.4 ± 1.5%), the subchondral trabecular bone density *ρ_B_* was 1.44 ± 0.01 g/cm^3^, and the subchondral trabecular bone compressive strength *S* was 22.9 ± 1.2 MPa. After the MSC-Scaffold embedding, the BA/TA value at the level just below the reference plane was 67.9% (the corresponding calculated subchondral trabecular bone density was 1.58 g/cm^3^ and the subchondral trabecular bone compressive strength was 37.7 MPa), so the change between the highest BA/TA values (at the level just below the reference plane) was the lowest in the study group 18.2 p.p. The changes in BA/TA value for the patient (evaluated from the best fit curve Figure 6) then decreased to ca. 10.0% at the level 1 mm (decrease rate 8.2% per 1 mm), reaching ca. 6.0% at the level 2 mm (decrease rate 4.0 p.p. per 1 mm), and ca. 3.5% at 3 mm (decrease rate 2.5 p.p. per 1 mm), respectively. The subchondral trabecular bone relative densification at the levels 0.5 mm and 1 mm was by 8.8% and 7.4%, respectively, while the relative change in the compressive strength of subchondral trabecular bone at the levels 0.5 mm and 1 mm was by 60.3% and 47.1%, respectively. The increase in the density of the subchondral trabecular bone below the embedded MSC-Scaffold was the lowest in the whole group and amounted to 1 mm. The relative change of subchondral trabecular bone density *ρ_B_* and compressive strength in the case of this patient was the lowest, and did not exceed 2.8% and 17.1%, respectively, at the level 1.5 mm, as well as at the lower levels. In this patient, the duration of symptoms was 2 years. X-ray indicated the 3 stage of the KL scale. Despite the pain (VAS 7), the patient moved using the elbow crutches. This case is the most unclear. In this patient, the increase in density was the lowest, although the initial density was not the lowest. When analyzing BA/TA points and changes, the initial low bone quality caused greater bone density (at 1 mm). It may indicate the presence of additional factors that reduce bone density. The degenerative changes affected only the right side. This patient was diagnosed with low bone density, probably caused by a significant relief of the affected limb one-sided degenerative changes. An additional factor in the patient that may affect the difference in results is obesity (BMI = 33.2).

Based on the above analysis of patients, it can be summarized that the highest (approx. 14%) density of subchondral trabecular bone directly under the MSC-Scaffold was obtained in patients (patient 1 and patient 2) with higher baseline BA/TA and the calculated bone density. The high baseline bone quality in these patients is likely due to a normal baseline bone density compared to other authors in OA [24]. Differences in the spatial extent of subchondral trabecular bone densification due to the MSC-Scaffold prototype initial embedding between these cases can be noticed, with a definite advantage in the male patient 1 (2.5 mm vs. 1.5–2 mm).

In all patients, the change in BA/TA 1 mm below the baseline was similar and ranged from 12.5–16.3 p.p. Interestingly, the highest change in density at the level of 1 mm was obtained in the patient with the lowest initial density (patient 3). 

The main complications of contemporary total hip resurfacing arthroplasties, in which the femoral component of the endoprosthesis is fixed with cement, due to the extensive penetration of cement into the periprosthetic bone, are aseptic loosenings and femoral neck fractures. One of the advantages of the proposed new technique of fixation of femoral components of THRA endoprostheses through the MSC-Scaffold is that it is entirely cementless. The other advantage is that in the MSC-Scaffold biomimetism the spikes of the scaffold mimic the interdigitations of subchondral bone, providing the nearly uniform transfer of mechanical loads from the femoral component of the THRA endoprosthesis to the subchondral trabecular bone of the femoral head. The performed pilot study of the micro-CT assessment and analyses of the MSC-Scaffold embedding in the human femoral subchondral heads of patients with OA determined the range of effect of the subchondral trabecular bone densification, taking into account different initial bone density. The found densification of the subchondral trabecular bone of the femoral head caused by the MSC-Scaffold embedding results in the increase in the mechanical strength of this bone. For the cementless innovative resurfacing THA (THRA), based on the here obtained results, we can expect that the improved mechanical properties of bone below the MSC-Scaffold condition the initial stability of femoral component of THRA endoprosthesis. The limitation of the presented innovative technique is the manufacturing of the MSC-Scaffold prototype in selective laser melting (SLM) technology, which requires considerable investments. The limitation of the study was the small sample size (four femoral head specimens from OA patients). Our study also did not refer to the control group. This was due to the difficulty of finding healthy femoral head bone specimens in the appropriate age and gender categories. Further research on this topic is needed to verify the pilot study results here obtained.

## 5. Conclusions

For the first time, the micro-CT assessment of the subchondral trabecular bone relative area and density in the femoral heads, removed during THA from OA patients before and after mechanical embedding of the MSC-Scaffold prototype for THRA endoprostheses into these heads, was carried out.

The embedding of the MSC-Scaffold in subchondral trabecular bone causes the change in its relative area (BA/TA) ranging from 18.2 to 24.7% (translating to the calculated density *ρ_B_* relative change from 11.1 to 14.4%, and the predicted compressive strength *S* relative change from 75.3 to 122.7%) regardless of its initial density (before the embedding).

The subchondral trabecular bone densification due to the MSC-Scaffold initial embedding gradually decreases with the increasing distance from the apexes of the MSC-Scaffold’s spikes, while the spatial extent of the subchondral trabecular bone densification ranged from 1.5 to 2.5 mm.

It can be suggested (despite the limited number of examined femoral head specimens) that the size of the influence of the MSC-Scaffold initial embedding on subchondral trabecular bone may be a factor in its maintenance. It seems that the deeper this effect of the subchondral trabecular bone densification, the better the strength of subchondral trabecular bone, and consequently, the better post-operative embedding of the MSC-Scaffold in the bone should be expected. The increased strength of subchondral trabecular bone can prevent the possible migration of implant during the postoperative limb loading.

## Figures and Tables

**Table 1:** Characteristics of the patients; KL Kellgren-Lawrence radiographic stage of OA, VAS Visual Analogue Scale.

Patient No.	Age	Sex	BMI	Side	Opposite Side OA	Duration of Pain [years]	Orthopedic Supports	KL	VAS
1	72	M	27.5	R	yes	5	orthopedic walker	3	6
2	54	F	24.0	R	yes	1	without orthopedic supports	4	7
3	68	F	28.8	R	yes	2	elbow crutches	3	7
4	51	M	33.2	R	no	2	elbow crutches	4	8

**Table 2:** Subchondral trabecular bone relative area (BA/TA) values in femoral heads from patients with OA measured in micro-CT before and after embedding of the MSC-Scaffold (MSCS), the percentage point changes in BA/TA values, the calculated corresponding subchondral trabecular bone densities *ρ_B_*, and subchondral trabecular bone compressive strength *S,* before and after embedding of the MSC-Scaffold, and changes in subchondral trabecular bone densities *ρ_B_* and subchondral trabecular bone compressive strength *S*; * spatial extent of subchondral trabecular bone densification due to the MSC-Scaffold prototype initial embedding [mm].

Patient No.		Levels Below Reference Plane [mm]									Mean ± SD
		0.0	0.5	1.0	1.5	2.0	2.5	3.0	3.5	4.0	
1	BA/TA before MSCS embedding [%]	58.4	56.1	57.3	58.4	57.3	56.5	57.2	56.9	57.9	57.3 ± 0.8
	BA/TA after MSCS embedding [%]	83.0	72.2	72.1	68.8	68.5	67.1	62.3	62.3	62.4	
	Change in BA/TA [%]	24.7	16.1	14.8	10.4	11.2	10.6	5.0 *	5.4	4.5	
	*ρ_B_* before MSCS embedding [g/cm^3^]	1.50	1.48	1.49	1.50	1.49	1.48	1.49	1.48	1.49	1.49 ± 0.01
	*ρ_B_* after MSCS embedding [g/cm^3^]	1.71	1.61	1.61	1.58	1.58	1.57	1.53	1.53	1.53	
	*ρ_B_* relative change [%]	14.3	9.0	8.3	5.6	6.3	6.1	2.9	3.1	2.5	
	*S* before MSCS embedding [MPa]	28.7	26.7	27.8	28.7	27.8	27.1	27.7	27.4	28.3	27.8 ± 0.7
	*S* after MSCS embedding [MPa]	54.1	42.0	41.9	38.5	38.3	36.9	32.2	32.3	32.4	
	*S* relative change [%]	88.6	57.5	51.0	34.3	37.7	36.3	16.3	17.8	14.3	
2	BA/TA before MSCS embedding [%]	55.1	54.1	52.0	53.2	52.2	52.0	51.9	52.7	52.8	52.9 ± 1.1
	BA/TA after MSCS embedding [%]	79.7	66.1	65.2	59.3	55.9	53.5	53.2	54.8	52.9	
	Change in BA/TA [%]	24.6	12.0	13.2	6.0	3.7 *	1.4	1.3	2.1	0.1	
	*ρ_B_* before MSCS embedding [g/cm^3^]	1.47	1.46	1.44	1.45	1.44	1.44	1.44	1.45	1.45	1.45 ± 0.01
	*ρ_B_* after MSCS embedding [g/cm^3^]	1.68	1.56	1.55	1.50	1.47	1.45	1.45	1.47	1.45	
	*ρ_B_* relative change [%]	14.4	6.9	7.5	3.3	1.8	0.6	0.6	1.5	0.1	
	*S* before MSCS embedding [MPa]	25.9	25.0	23.3	24.3	23.5	23.3	23.2	23.8	23.9	24.0 ± 0.9
	*S* after MSCS embedding [MPa]	50.3	35.9	35.0	29.5	26.5	24.5	24.3	25.6	24.1	
	*S* relative change [%]	94.3	43.2	50.1	21.3	12.9	5.0	4.5	7.3	0.5	
3	BA/TA before MSCS embedding [%]	40.3	41.9	39.6	40.7	38.7	41.7	41.0	41.4	39.9	40.6 ± 1.0
	BA/TA after MSCS embedding [%]	62.9	58.1	55.9	52.6	48.5	42.8	42.1	42.0	42.6	
	Change in BA/TA [%]	22.6	16.2	16.3	11.9	9.8 *	1.1	1.1	0.6	2.8	
	*ρ_B_* before MSCS embedding [g/cm^3^]	1.34	1.36	1.34	1.35	1.33	1.35	1.35	1.35	1.34	1.34 ± 0.01
	*ρ_B_* after MSCS embedding [g/cm^3^]	1.53	1.49	1.47	1.45	1.41	1.36	1.36	1.36	1.36	
	*ρ_B_* relative change [%]	14.0	9.9	10.0	7.7	6.1	0.4	0.9	0.6	1.6	
	*S* before MSCS embedding [MPa]	14.7	15.8	14.2	15.0	13.7	15.7	15.2	15.4	14.5	14.9 ± 0.7
	*S* after MSCS embedding [MPa]	32.8	28.5	26.5	23.8	20.6	16.4	15.9	15.9	16.3	
	*S* relative change [%]	122.7	80.2	86.2	58.9	49.9	4.8	4.8	4.8	12.8	
4	BA/TA before MSCS embedding [%]	49.7	50.0	52.2	54.0	53.1	51.9	51.4	50.9	49.9	51.4 ± 1.5
	BA/TA after MSCS embedding [%]	67.9	65.0	64.7	59.0	57.4	55.3	55.0	53.7	53.9	
	Change in BA/TA [%]	18.2	15.0	12.5	4.9 *	4.3	3.4	3.6	2.8	4.0	
	*ρ_B_* before MSCS embedding [g/cm^3^]	1.42	1.43	1.44	1.46	1.45	1.44	1.44	1.43	1.42	1.44 ± 0.01
	*ρ_B_* after MSCS embedding [g/cm^3^]	1.58	1.55	1.55	1.50	1.49	1.47	1.47	1.46	1.46	
	*ρ_B_* relative change [%]	11.1	8.8	7.4	2.8	2.7	2.0	2.3	1.9	2.5	
	*S* before MSCS embedding [MPa]	21.5	21.8	23.5	25.0	24.2	23.2	22.8	22.4	21.6	22.9 ± 1.2
	*S* after MSCS embedding [MPa]	37.7	34.9	34.5	29.2	27.8	26.0	25.8	24.7	24.8	
	*S* relative change [%]	75.3	60.3	47.1	17.1	15.1	12.0	13.0	10.2	14.9	

## Data Availability

The data presented in this study are available on request from the corresponding author.
